# Prediction of some quality properties of rice and its flour by near‐infrared spectroscopy (NIRS) analysis

**DOI:** 10.1002/fsn3.2086

**Published:** 2020-12-25

**Authors:** Nasrollah Fazeli Burestan, Amir Hossein Afkari Sayyah, Ebrahim Taghinezhad

**Affiliations:** ^1^ College of Agriculture and Natural Resources University of Mohaghegh Ardabili Ardabil Iran; ^2^ Faculty of Agriculture and Natural Resources University of Mohaghegh Ardabili Ardabil Iran; ^3^ Moghan College of Agriculture and Natural Resources University of Mohaghegh Ardabili Ardabil Iran

**Keywords:** Amylose content, NIR spectroscopy, rice quality, setback viscosity

## Abstract

The measurement of different quality properties requires particular tools and chemical materials, most of which are time‐using. The present research was accomplished to survey the possibility of using NIRS (870–2450 nm) to predict the amylose content (AC), protein content (PC), breakdown (BDV), and setback viscosity (SBV) of white rice (*Khazar* variety) and its flour. Determination coefficients of calibration models to flour samples of AC, PC, BDV, and SBV generated by the partial least‐squares (PLS) regression were obtained as *R* ^2^_cal_ ≥ .85 and *R* ^2^_pre_ ≥ .80. Root mean square error of calibration (RMSEC) was calculated as 0.393, 0.07, 2.55, and 1.33, respectively. Similarly to grain samples, were obtained as *R* ^2^_cal_ ≥ .88 and *R* ^2^_pre_ ≥ .71 for calibration and prediction. RMSEC was measured as 0.303, 0.27, 2.59, and 3.11, respectively. NIRS has the potential to be used as a quick technique for predicting the quality attributes of kernel specimens.

## Graphical abstract

Determination coefficients of calibration models to flour samples of AC, PC, BDV, and SBV generated by the PLS regression were obtained as *R* ^2^_cal_ ≥ .85 and *R* ^2^_pre_ ≥ .80. Root mean square error of calibration (RMSEC) was calculated as 0.393, 0.07, 2.55, and 1.33, respectively, and to grain samples, was obtained as *R* ^2^_cal_ ≥ .88 and *R* ^2^_pre_ ≥ .71 for calibration and prediction. RMSEC was measured as 0.303, 0.27, 2.59, and 3.11, respectively.

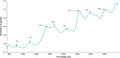

## 1 INTRODUCTION

The commercial value of rice is largely determined by the quality characteristics of the milled rice (Vithu et al., 2016). Quality indices such as AC, temperature of gelatinization, consistency of gel, and pasting properties play a very important role in the final quality of rice (Ferreira et al., 2017). Bao et al. (2007) studied on the determination of quality indices of rice by NIR, and they reported that a direct measurement is time‐consuming and expensive; therefore, a rapid predictive method based on NIRS is useful for the measurement of these quality indices. Compared to conventional analysis procedures, NIRS has numerous advantages including simplicity and promptitude of sample preparation. A number of researches for example (Osborne, 2006) have reported the use of calibration models for determining the PC of rice. Siriphollakul et al. (2017) reported that using the NIRS is possible to predicting AC of rice. Natsuga and Kawamura (2006) studied on determining the quality attributes of rice using NIRS. The results of the PLS regression model used to predict the content of moisture (MC) and PC of brown rice showed the coefficients of correlation (*R*) of .98 and .96, respectively, and for processed rice (milled rice), breakdown and peak viscosity showed the *R* of .91 and .87, respectively. NIRS has been reported to be used for determining the quality attributes and assessing its quality.

Chen et al. (2008) studied on the determination of the PC combination of rice starch using the NIRS (1100–2500 nm), and they reported that NIRS has the potential to serve as a quick procedure for predicting the PC combination. Siriphollakul et al. (2015) also studied on the pasting attributes of paddy rice samples by NIR (1400–2400 nm) analysis and reported that the expanded models of SBV and BDV provided good prediction consequences with *R* (.81–.96). Bao et al. (2001) studied on the utilization of NIRS for measuring several quality parameters of rice, amylase content (AC), pasting parameters of setback (SB), breakdown (BD), and pasting temperature (PT), and reported it as the most appropriate model for predicting the AC, BD, SB, and PT (.88 < *R* ^2^ < .92).

The quality indices of white rice are useful for the consumers in terms of marketing of this product. Quality analysis of rice, which is usually used in food industry, is time‐consuming and highly expensive due to requiring special testing instruments. This study was conducted to use NIR spectroscopy a quick and nondestructive techniques to determine the quality of rice, through focusing on the chemical and physicochemical properties. As mentioned earlier, the present study was conducted to expand NIRS calibration models for predicting the AC, PC, BDV, and SBV of rice and its flour specimens on the common Iranian rice (*Khazar* variety).

## 2 MATERIALS AND METHODS

### 2.1 Row specimen provision

The bulk samples were prepared in sufficient values from the common Iranian variety of rice namely *Khazar* obtained directly from the field of Rice Research Institute located in Rasht, northern Iran. The initial MC was determined by placing three samples (weighing 15 g) into a laboratory oven with a temperature of 130°C for 24 hr (Li et al., 2014). Moisture content of the sample was equal to 15.3% (w.b.). Then, to reduce MC, the paddies were spread on a floor in thin layers and dried by a laboratory drier at a temperature of 35°C. After crushing their paddy husk, they were milled using a UDY cyclone miller under the same condition.

### 2.2 Analyses of spectroscopic

Spectrum of kernel specimens weighing almost 5 g was recorded using a NIR spectrometer (Ocean Optics NIR Quest 256, American) in the Laboratory of Optic Physics located in the Research Institute of Color Science and Technology. A rotating cup was used for all of samples, that is, rice grains and its flour. Spectrometer is equipped with the InGaA (*Indium Gallium Arsenide*) detector, and inflammatory lamps (incandescent lamps) were used as a light source. The samples were subjected to the spectrometer by contacting the inner part of a cylindrical probe with the lamps̓ radiation, so that the light was reflected through the probe by an optical fiber. Reflected light from the surface of the samples was displayed by the Ocean Optics Spectra Suite software in different measurement modes. Reflectance (R) readings at 6.5‐nm increments (Siriphollakul et al., 2017) were gathered over a NIR wavelength range of 870–2,450 nm and were recorded as log (1/R). For the chemometric analysis, three repeats for each specimen were accomplished and averaged, along with sample repacking.

### 2.3 Reference analyses

In this study, some chemical properties including AC, PC, and the starch physicochemical properties including BDV and SBV were assessed. The AC was determined through colorimetry at the wavelength of 620 nm by forming an iodine–starch complex (Juliano, 1971). The PC of the rice was determined by the Kjeldahl procedure with a factor of nitrogen conversion (5.95) (Xu et al., 2008). To measure the physicochemical attributes of the flour, specimens were milled using a UDY cyclone miller and then were passed through a 100‐mesh sieve. Then, 3 g of each sample was separated. After that, 25 ml of distilled water was added to the separated samples. Then, the resultant mixtures were placed inside the metal cylinder of Rapid Visco Analyzer (RVA‐3D; Newport Scientific) (Kesarwani et al., 2016). The results of the test are presented as a curve showing the changes in the viscosity of the samples at various temperatures of cooking.

### 2.4 Chemometric analyses

Before the development of calibration model, the reference amount outliers (5 samples for the rice grains and 3 specimens for its flour) were recognized by the principal component analysis (PCA). The outliers limited to the excess of outliers were removed. The validation and calibration processes accomplished for rice kernel and its flour spectrums then are called the rice and its flour set, respectively. All sample spectrums were separated into two subsets including calibration and external validation (Table 1). Preprocessing of spectrums was carried out and calibration model was expanded relying on regression of PLS in accordance with a specimen test set, using the software for multivariate analysis (The Unscrambler 9.8; CAMO). Thus, 81 samples for rice grain and 84 samples for rice flour were comprised in the calibration set, and also, 28 and 27 specimens were comprised in the validation set, respectively. Specimen sets of the calibration and validation are shown in Table 1. Then, set of the calibration was used to produce the calibration models with estimated amounts via the reference and spectral data, while the validation set was used to appraise the resulting patterns (Chen et al., 2008). All sample spectra were preprocessed by multiplication scatter correction (MSC) and first derivative (D_1_). To avoid enhancing the noise, spectra were first smoothed, which was done by using the Savitzky–Golay (SG) algorithm (Sinija & Mishra, 2011).

**TABLE 1:** Number of samples, minimum, maximum, means, and standard deviation (*SD*) in two calibration and validation groups of rice and its flour

Parameter	Calibration					Validation				
	No	Min	Max	Mean	*SD*	No	Min	Max	Mean	*SD*
Rice flour										
AC	84	20.89	22.24	21.56	1.02	27	20.68	22.56	21.53	1.07
PC	84	7.5	10	9.7	0.91	27	8.6	9.9	9.7	0.59
BDV	84	67	105	71.15	12.97	27	70.7	105	71.15	18.2
SBV	84	124	145.33	131.68	6.88	27	125	145.33	134.74	8.09
Milled rice										
AC	81	21.21	21.89	21.56	0.88	28	21.01	21.99	21.56	0.89
PC	81	6.8	10.35	9.7	1.19	28	7.2	10.5	9.69	1.12
BDV	81	57	120	71.15	20.51	28	71.15	137.6	92.15	23.77
SBV	81	130.54	161.35	145.33	11.52	28	130.25	161.54	145.32	10.82

Abbreviations: AC, amylose content; PC, protein content; BDV, breakdown viscosity; SBV, setback viscosity.

In modeling of calibration, prediction ability of the model's was decided using a leave‐one‐out cross‐validation (CV) and via coefficient of determination (*R* ^2^_cal_) quantified between measured values from each observation and predicted values from cross‐validation, as well as calculating the RMSECV. Results of prediction were appraised by computing the RMSEC, determination coefficient (*R* ^2^_pre_) between reference and predicted amounts, and residual predictive deviation (RPD) (Hassan et al., 2015; Siriphollakul et al., 2017). The RPD (the proportion of standard deviation (*SD*) to RMSECV) was used to confirm the calibration model precision. The model with the highest RPD value is the admissible model for analysis of quantitative (Hassan et al., 2015). Optimal PLS factor was elected conforming to the lowermost RMSECV value (Hassan et al., 2015).

## 3 RESULTS AND DISCUSSIONS

### 3.1 Quality parameters of rice

The ranges, means, and *SD* for each of the starch quality parameters in subsets of the calibration and validation are shown in Table 1. AC was found to be ranged from 20.89% to 22.24% among the samples prepared from *Khazar* rice variety. AC has been reported as the main factor influencing the physicochemical attributes of rice starch (Sodhi & Singh, 2003). PC was found to be ranged from 6.8% to 10.5% among the samples prepared from *Khazar* rice variety. PC is the second most plentiful constituent of processed rice after starch and it influences the physical attributes of cooked rice, so that the higher the PC, the harder and less adhesive the rice grains become upon boiling (Kadan et al., 1997). High amounts in BDV and low amounts in SBV are indicative quality of high cooking because the cooked rice neither retrogrades nor becomes firm upon cooling (Allahgholipour et al., 2006). *Khazar* rice variety with low breakdown viscosity and high setback viscosity becomes firm after cooking (Asante et al., 2013).

### 3.2 Features of NIRS spectra for rice flour and grain samples

**FIGURE 1:**
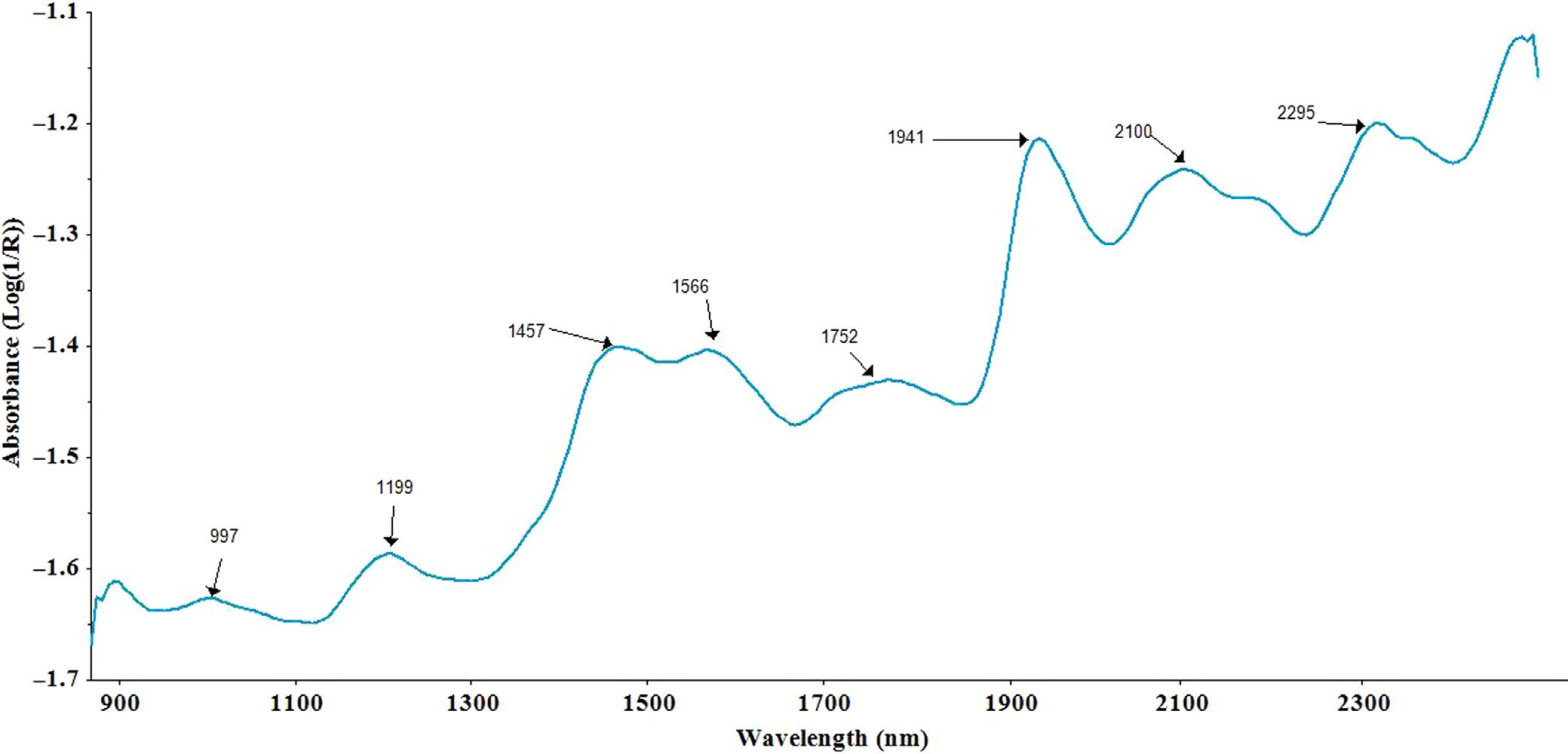
Row NIR spectra of rice flour samples

The spectra of rice and its flour specimens are represented in Figure 1 showing the spectrum signals before pretreatment.

As shown in Figure 1, bands of the main absorption were observed at wavelengths of 997, 1,199, 1,457, 1,566, 1,752, 1,938, 2,100, 2,180, and 2,295 nm. Similarly, for grain samples, bands were observed at wavelengths of 998, 1,199, 1,456, 1,573, 1,750, 1,940, 2,094, and 2,200 nm. NIRS (870–2450 nm) showed at wavelengths of 997 and 998 nm attributing to (the O‐H or NH2 second overtone), 1,199 nm (the NH2 or O‐H overtone of second), 1,457 and 1,566 and 1,573 nm (the O‐H and N‐H first overtone), 1752 nm (the C‐H first overtone), 1,938, 2,094, 2,100, and 2,180 nm (the O‐H and N‐H combinations), and 2,295 nm (the C‐H combinations). Similar observations were reported by Chen et al. (2018) and Osborne (2006).

### 3.3 PLS models and results

The results obtained from the calibration and validation of PLS regression models developed from rice and its flour samples for AC, PC, BDV, and SBV are shown in Table 2. Starred values also highlight the best models in these tables. A good model for predicting should prepare a high *R* ^2^, as well as low values of RMSECV and RMSEC (Siriphollakul et al., 2017). As shown in Table 2, for rice flour samples, the best PLS models were obtained from the SG+D_1_, SG+MSC, SG+MSC, and nonprocessed, respectively, for AC, PC, BDV, and SBV prediction, along with pretreated absorbance spectra in the NIR range of 870–2450 nm, with *R* ^2^_val_ of .79, .988, .95, and .945, respectively, indicating relatively good performance of the models. Similar observations were reported by Osborne et al. (1993). Prediction results also revealed low values of RMSEC for AC, PC, BDV, and SBV (0.393%, 0.07%, 2.55%, and 1.33%, respectively). Regarding the RPD, PLS models furnished amounts greater than 2.27, in particular for AC, PC, BDV, and SBV (2.27, 5.96, 6.17, and 4.97, respectively), which was verified by the data observed in other studies (Chen et al., 2008; Hassan et al., 2015). An RPD from 2 to 10 is considered useable and reliable (Fearn, 2002) and from 2.5 to 3 or above corresponds to good and superfine prophecy precision (Nicolai et al., 2007). The amounts of RMSEP were comparatively low, meaning the models could possibly predict the aimed attributes. The present research showed comparatively high *R* ^2^_pre_ and low RMSEC and RMSECV amounts.

**TABLE 2:** The results of the calibration and validation of PLS regression models developed from rice and its flour samples

Parameter	Pretreatment	Factors	Calibration			Validation			
			*R* ^2^_cal_	RMSEC	SEC	*R* ^2^_pre_	RMSECV	SECV	RPD
Flour samples									
AC	None	6	.884	0.388	0.404	.781	0.482	0.485	2.22
	SG+D_1_ ^a^	**7**	**.851**	**0.393**	**0.395**	**.79**	**0.472**	**0.475**	**2.27**
	SG+MSC	7	.85	0.395	0.398	.784	0.479	0.481	2.23
PC	None	6	.984	0.113	0.114	.977	0.139	0.14	4.25
	SG+D_1_	5	.978	0.136	0.137	.965	0.173	0.173	3.41
	SG+MSC^a^	**7**	**.994**	**0.07**	**0.07**	**.988**	**0.099**	**0.099**	**5.96**
BDV	None	7	.956	2.71	2.73	.935	3.336	3.356	5.45
	SG+D_1_	7	.962	2.506	2.52	.944	3.098	3.11	5.87
	SG+MSC^a^	**8**	**.961**	**2.55**	**2.57**	**.95**	**2.948**	**2.96**	**6.17**
SBV	None^a^	**7**	**.962**	**1.33**	**1.34**	**.945**	**1.628**	**1.64**	**4.97**
	SG+D_1_	7	.956	1.44	1.45	.934	1.786	1.79	4.52
	SG+MSC	6	.958	1.41	1.42	.94	1.70	1.71	4.76
Grain samples									
AC	None	7	.868	0.318	0.32	.753	0.441	0.44	2.02
	SG+D_1_ ^a^	**7**	**.881**	**0.303**	**0.305**	**.802**	**0.395**	**0.398**	**2.25**
	SG+MSC	7	.872	0.314	0.316	.755	0.44	0.442	2.02
PC	None	8	.916	0.344	0.346	.75	0.6	0.6	1.87
	SG+D_1_	8	.944	0.28	0.282	.797	0.541	0.54	2.07
	SG+MSC^a^	**8**	**.948**	**0.27**	**0.272**	**.816**	**0.51**	**0.519**	**2.2**
BDV	None	8	.983	2.66	2.67	.952	4.55	4.58	4.89
	SG+D_1_ ^a^	**8**	**.984**	**2.59**	**2.61**	**.954**	**4.428**	**4.45**	**5.03**
	SG+MSC	7	.957	4.238	4.26	.90	6.54	6.58	3.4
SBV	None^a^	**8**	**.927**	**3.11**	**3.13**	**.793**	**3.306**	**5.34**	**3.27**
	SG+D_1_	8	.936	2.919	2.94	.844	4.61	4.64	2.35
	SG+MSC	8	.935	2.929	2.95	.802	5.189	5.22	2.09

Abbreviations: None, raw; SG+D_1_, Savitzky–Golay+first deviation; SG+MSC, Savitzky–Golay+multiplication scatter correction; *R* ^2^_cal_ and *R* ^2^_pre_, coefficient of determination for calibration and prediction; SEC, standard error of calibration; SECV, standard error of cross‐validation; RMSEC, root mean square error of calibration; RMSECV, root mean square error of cross‐validation.

^a^ Starred values’ highlight the best models developed.

As shown in Table 2, for rice grain samples, the best PLS models were obtained from the SG+D_1_, SG+MSC, SG+D_1_, and nonprocessed, respectively, for AC, PC, BDV, and SBV prediction. Also, pretreated absorbance spectra were appeared, with *R* ^2^_val_ of .802, .816, .954, and .793, respectively. Prediction results also disclosed low values of RMSEC for AC, PC, BDV, and SBV (0.303%, 0.27%, 2.59%, and 3.11%, respectively). Regarding the RPD, PLS models furnished amounts greater than 2.2, in particular for AC, PC, BDV, and SBV (2.25, 2.2, 5.03, and 3.27, respectively). Among them, higher RPD values (4.97 and 3.27) were mostly obtained for SBV of the rice and its flour samples, respectively, which are consistent with the previous reports by Siriphollakul et al. (2015). NIRS calibration for AC, PC, BDV, and SBV gave favorable results for milled rice and its flour (Bao et al., 2007). For AC, no matter what specimens are used, good results are always obtained (Bao et al., 2001). In this study, because the white rice (*Khazar* variety) skin is not transparent. Thus, the rice grain is not much different from its flour. *R* ^2^_pre_ for AC using white rice was poorer than those using their corresponding flour samples, while *R* ^2^_pre_ for BDV using flour (.86) was poorer than those using their corresponding white rice (.89) samples. Starch quality indexes, such as AC, PC, and SBV, could be predicted with enough precision using NIRS based on flour spectrum, whereas only BDV could be predicted with enough precision based on grain spectrum.

In this research, the absorption peaks at wavelengths of 1,566 and 2,100 can be attributed to the SBV. These findings are in line with the findings reported by Osborne (2006) and Siriphollakul et al. (2015. Also, the absorption peak at wavelength of 997 nm could be correlated with the BDV. These findings are consistent with Natsuga and Kawamura (2006); Onda et al. (1994); and Osborne et al. (1993). Bands of the main absorption at wavelengths of 1,199, 1,750, 2,094, and 2,295 nm can be attributed to the PC, which is similar to the results reported by Chen et al. (2008); Onda et al. (1994); and Osborne et al. (1993). The figure obtained by NIRS for rice flour in spectra range of 1,100–2,500 nm (Chen et al., 2008) is similar to that presented in Figure 1 in this study. Osborne et al. (1993) reported that starch and AC are typically sensitive to wavelengths of 1,900 and 2,100 nm. Also, Bao et al. (2007) reported that AC and BDV at wavelengths of 1,932 and 2,292 nm had *R* ^2^ of .95% and .65%, respectively.

**FIGURE 2:**
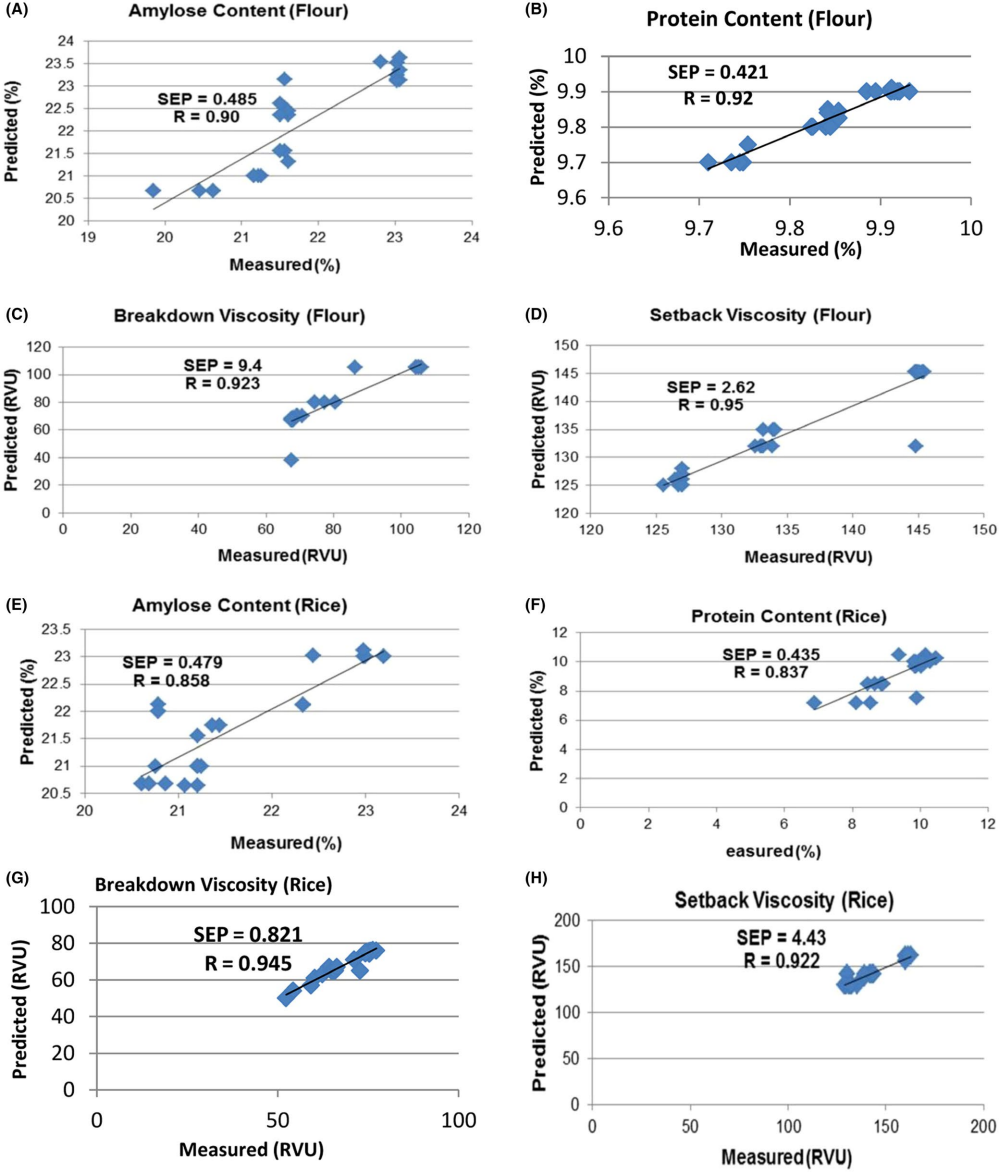
Scatter plots of predicted and measured amounts of quality attributes by model of PLS for validation set of rice and its flour samples

Figure 2a–h show the scatter plots related to the predicted and measured values of the chemical and physicochemical properties for rice flour and grain samples in the validation set, respectively. Influence on the model was measured in terms of relative leverage. All samples have leverages between 0.02 and 0.15. It depends on the data scatter. Therefore, the raw data were investigated and the high‐leverage sample was removed from the model. Also, regression equations related to the chemical and physicochemical properties are shown in Table 3. Dots scattered close to the target line (diagonal line) indicated that NIRS could predict all the chemical and physicochemical properties accurately or predicted values were highly correlated with the evaluated amounts.

**TABLE 3:** Regression equations of chemical and physicochemical properties by PLS model for validation set of rice and its flour samples

Parameter		Equivalent		*R* ^2^
Flour samples				
AC		Y = 0.9785x + 0.8267		.80
PC		Y = 0.8236x + 1.7027		.849
BDV		Y = 1.0636x−5.01117		.858
SBV		Y = 0.9831x + 1.5793		.90
Rice samples				
AC		Y = 0.8814x + 2.6467		.7363
PC		Y = 0.9806x−0.0022		.7126
BDV		Y = 0.8968x + 9.9288		.893
SBV		Y = 0.8939x + 15.568		.85

## 4 CONCLUSION

Considering that the conventional methods of rice's quality assessment usually used in food industry laboratories are time‐consuming and expensive due to requiring special testing instruments, nondestructive measurement of chemical and physicochemical properties of rice flour and grain samples is possible using the NIRS in different varieties with considerable accuracy. The results of this study showed that the suggested technique had satisfactory performance in predicting the AC, PC, BDV, and SBV, while the validity of the calibration models was statistically tested. Prediction accuracy of quality parameters for rice and its flour samples was obtained as *R* ^2^ ≥ .80 and *R* ^2^ ≥ .71, respectively. The results of the present research demonstrated that the use of NIRS is appropriate for predicting the quality attributes of rice and its flour.

## Supporting information

Data S1

Data S2
